# 

**Published:** 1921-03-26

**Authors:** 


### The Hospital

The Workers' Newspaper of Administrative Medicine and Institutional Life, Administration, National Insurance and Health.

CONTENTS.

**Interim Report of the Cave Committee** ... ... 575
**The League of Mercy and Gifts in Kind** ... 576
**Hospital and Institutional News** ... ... 577
Death of Mr. Henry Bonham-Carter—Scottish Board of Health—The Saturday Fund's Example—Saturday and Sunday Funds in Norwich—Adopt a Hospital!—Dispensaries and Unemployment—Hospital Medical Appointments—The Middle Classes Union and Hospitals—Misunderstood—Paying Patients in Infirmaries—The Hospital Guild in Birmingham—A Chairman and Approved Societies—Never in Debt—The Bombardment of Cameron Hospital—Mr. E. J. Thomas's Criticism—A Friendly Society Members' Suggestion—St. Thomas's Appeal.
**The Case against Maintenance Charges** ... ... 580
**Dried Milk...** ... ... ... ... 581
II. Practical Uses and Advantages.
**Conditions of Childbirth in India** ... ... ... 582
**Dutch Anti-Tuberculosis Work** ... ... ... 583
A Carefully Thought-out System.

CONTENTS

**Six Papers by Lord Lister** ... ... ... ... 584
**The Institutional Library** .. ... ... ... 585
**The Public Well-Being** ... ... ... ... 587
Housing and Child Welfare—Multiplication of the Unfit—Health Dangers in Unemployment—To Eat Well or to Live Ill—Anonymity for Young Criminals.
**A Family's Great Record** ... ... ... 591
Fielding Johnson of Leicester.
**The Editor's Letter-Box** ... .. ... ... 592
Unemployment Insurance: Minister's Decision—The Almoner Question—Should Nurses Smoke?
**The Matrons' and Sisters' Department** ... ... 593
The Pay of the Nurse: III. The Value of Board and Lodging.
"The Coming of the Mob."
**Round the Hospitals** ... ... ... ... 594
**Voluntary Hospitals Committee** ... ... ... 597
**The College of Nursing (Limited by Guarantee)** 600

